# Direct Inhibition of RNAse T2 Expression by the HTLV-1 Viral Protein Tax

**DOI:** 10.3390/v3081485

**Published:** 2011-08-18

**Authors:** Nicholas Polakowski, Hongjin Han, Isabelle Lemasson

**Affiliations:** Department of Microbiology and Immunology, Brody School of Medicine, East Carolina University, 600 Moye Boulevard, Greenville, NC 27834, USA; E-Mail: hanj@ecu.edu

**Keywords:** HTLV-1, Tax, RNase T2, repression, transcription

## Abstract

Adult T-cell leukemia (ATL) is one of the primary diseases caused by Human T-cell Leukemia Virus type 1 (HTLV-1) infection. The virally-encoded Tax protein is believed to initiate early events in the development of this disease, as it is able to promote immortalization of T-cells and transformation of other cell types. These processes may be aided by the ability of the viral protein to directly deregulate expression of specific cellular genes through interactions with numerous transcriptional regulators. To identify gene promoters where Tax is localized, we isolated Tax-DNA complexes from an HTLV-1-infected T-cell line through a chromatin immunoprecipitation (ChIP) assay and used the DNA to probe a CpG island microarray. A site within the *RNASET2* gene was found to be occupied by Tax. Real-time PCR analysis confirmed this result, and transient expression of Tax in uninfected cells led to the recruitment of the viral protein to the promoter. This event correlated with a decrease in the level of RNase T2 mRNA and protein, suggesting that Tax represses expression of this gene. Loss of RNase T2 expression occurs in certain hematological malignancies and other forms of cancer, and RNase T2 was recently reported to function as a tumor suppressor. Consequently, a reduction in the level of RNase T2 by Tax may play a role in ATL development.

## 1. Introduction

Human T-cell Leukemia Virus type 1 (HTLV-1) is a complex retrovirus that causes a fatal form of leukemia known as adult T-cell leukemia (ATL) [1]. This disease occurs through the clonal expansion and subsequent transformation of one or possibly a few HTLV-1-infected CD4+ T-cells, which are the primary target of the virus. The virally-encoded Tax protein is believed to be critically involved in the development of ATL. Tax has been shown to be oncogenic through its ability to transform murine fibroblasts in a Ras-dependent manner, immortalize primary T-cells, and cause tumors in transgenic mice [2]. Moreover, transgenic mice that express Tax specifically through the *LCK* promoter in the thymus exhibit some pathological conditions similar to those of ATL patients [3]. Overall, these effects are believed to arise from the ability of Tax to stimulate cell proliferation and inhibit apoptosis, while concomitantly promoting increased DNA damage and genome instability [4].

The deregulation of cellular processes by Tax is frequently caused by changes in gene expression. At the HTLV-1 promoter, Tax functions as a transcriptional activator through the formation of a highly stable complex with the transcription factor CREB and the coactivator p300/CBP [5]. In the context of cellular genes, Tax activates transcription indirectly through stimulation of NFκB and Akt signaling and directly through interactions with CREB and SRF [2]. Interestingly, the association of Tax with CREB on some promoters also inhibits transcription, potentially by blocking interactions between CREB and positive regulators [6,7]. Tax has also been found to repress transcription by binding directly to p300 and CBP and sequestering the coactivators away from activator complexes at cellular promoters [8]. This model defines how Tax classically inhibits transcriptional activation by basic helix-loop-helix factors [9,10], although separate mechanisms are also involved [11]. Overall, the direct effects of Tax on cellular gene expression are mediated by interactions between the viral protein and a large array of transcriptional regulators [12], as Tax alone does not bind DNA.

In lieu of the oncogenic properties of Tax, less than five percent of the HTLV-1-infected population develops ATL, with the disease normally occurring decades after the initial infection [1]. Therefore, environmental, hereditary and other factors may dictate whether Tax helps trigger the proliferation, survival and, ultimately, transformation of an infected T-cell. It is also possible that Tax itself may contribute to the low incidence of ATL, as Tax is known to induce apoptosis, an outcome that frequently predominates over its proliferative and pro-survival effects [13]. Similarly, Tax induces senescence in certain cell-types through the upregulation of the cyclin-dependent kinase inhibitors p21 and p27 [14,15]. Interestingly, results from a recent report demonstrate that a small fraction of the senescent cells can reenter the cell cycle, although it is unclear whether Tax is involved in this process [16].

Ribonuclease T2 (RNase T2) is known to play a role in senescence in plants and possibly mammalian cells [17,18]. This protein may be an important tumor suppressor, as it is located within a region of the long arm of chromosome six that undergoes deletion or rearrangement in many cancers, including some cases of ATL [19,20]. Although this classification has been challenged [21,22], results from many studies link RNase T2 expression to inhibited growth of immortalized and tumor cells [17,23–27]. Recently, a definitive tumor suppressor function for RNase T2 was characterized *in vivo* using a mouse model, indicating that inhibition of tumor growth by RNase T2 mainly occurs in the context of the tumor microenvironment [26].

In the current study, we found that Tax is recruited to the *RNASET2* gene. This interaction was identified using chromatin immunoprecipitated (ChIPed) DNA from MT-2 cells to probe a microarray and was confirmed using quantitative real-time PCR (qPCR) analysis of ChIPed DNA. In addition, Tax from an HTLV-1-infected cell nuclear extract bound to a region of the *RNASET2* gene in an immobilized template-binding assay. The association of Tax with this region repressed RNase T2 expression. Two Tax-interacting partners, Brm, which is one of the core subunits of the SWI/SNF complex, and histone deacetylase 1 (HDAC1), bound to the same region of the *RNASET2* gene as Tax. Given that these proteins appeared to associate with the gene independent of Tax, it is possible that they facilitate in the recruitment of the viral protein to the gene.

## 2. Results and Discussion

### 2.1. Tax Is Enriched at the RNASET2 Gene

**Figure 1.**
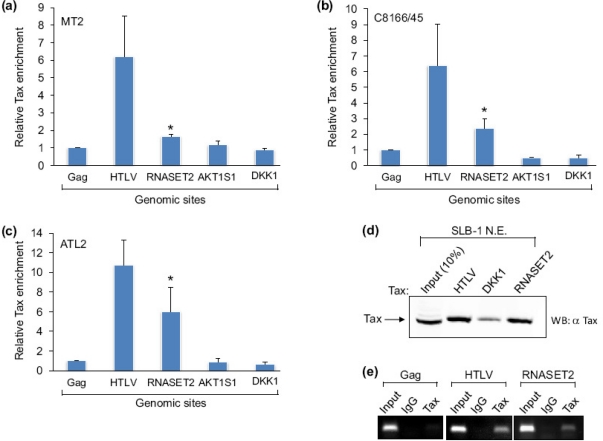
The association of Tax with the *RNASET2* gene. (**a**) Enrichment signals for Tax at the HTLV-1 promoter, the *RNASET2* gene (+1069 to +1153), the *ALT1S1* promoter and the *DKK1* promoter were determined by qPCR analysis of ChIP samples relative to the Tax signal at the *gag* site in MT-2 cells. The 2^CT(Input)−CT(IP)^ values obtained at each site were normalized to the value obtained at *gag*, which was set to 1. The graph shows data averaged from independent ChIP assays. Error bars show standard deviations. No standard deviation is shown for *gag*, as values at this site were set to 1 to normalize data among ChIP assays. The asterisk denotes a *p*-value of 0.015 for a two-tailed Student *t*-test. (**b**) Enrichment signals for Tax in C8166/45 cells (*n* = 4; *p* = 0.004). (**c**) Enrichment signals for Tax in ATL-2 cells (*n* = 4; *p* = 0.003). (**d**) The Western blot shows Tax from an SLB-1 cell nuclear extract retained on immobilized DNA templates that separately contained the HTLV-1, *DKK1* and *RNASET2* regions from −306 to −1, −539 to −244, and +1069 to +1545, respectively. (**e**) PCR analysis of ChIP samples shows specific Tax binding at the HTLV-1 promoter and *RNASET2* gene. Coimmunoprecipitation using preimmune rabbit serum (IgG) and amplification of the gag site were performed as negative controls. Each panel shows amplification of 1% of the total input chromatin (input).

Tax predominantly localizes to the nucleus where it is able to interact with several cellular transcription factors and other transcriptional regulators. These properties allow Tax to associate with specific DNA-bound protein complexes and influence gene expression. To identify genomic sites directly targeted by Tax, DNA fragments associated with protein complexes containing Tax were isolated by chromatin immunoprecipitation (ChIP) from the HTLV-1-infected T-cell line, MT-2, using a Tax-specific antibody. Following amplification and fluorescent labeling, ChIP DNA was used to probe a CpG island microarray, as CpG islands are frequently found within transcriptionally active regions of the genome. Through this approach we identified a region of the *RNASET2* gene (+1071 to +1366 with respect to the RNA start site) as a possible Tax-binding site. To verify this result, we quantified relative levels of Tax enrichment at various genomic sites through qPCR analysis of ChIP samples obtained from different HTLV-1-infected cell lines. In each cell line, the level of Tax enrichment at the *RNASET2* gene was compared with levels of enrichment at the HTLV-1 promoter (a known Tax-binding site) and within the proviral *gag* gene (negative for Tax). Two additional negative sites were also evaluated: the *AKT1S1* promoter, which did not exhibit Tax enrichment in the microarray analysis, and the *DKK1* promoter, which is indirectly regulated by Tax [28]. Surprisingly, although we originally identified the *RNASET2* site from the microarray analysis using MT-2 cells, Tax enrichment at this position was only slightly greater than at the three negative sites (Figure 1a). In contrast, Tax-binding to the *RNASET2* gene was more pronounced in C8166/45 and ATL-2 cells (Figures 1b,c). In all three cell lines, the HTLV-1 promoter exhibited the strongest signal for Tax consistent with the presence of multiple binding sites for Tax-containing complexes within this region of the DNA. It is important to note that small amounts of nonspecific protein-DNA complexes may co-purify with specific complexes in ChIP assays. This phenomenon leads to a background signal at negative sites that is detected by real-time PCR. Furthermore, in the case of the *gag* site, a fraction of the fragmented chromatin that is coimmunoprecipitated with Tax is longer than the 683 base pairs separating this position and the HTLV-1 promoter. Therefore, some chromatin may contain both sites even though Tax is bound to the HTLV-1 promoter.

We corroborated ChIP results *in vitro* using an immobilized template-binding assay in which we isolated protein complexes from an HTLV-1-infected cell nuclear extract using various DNA templates linked to Dynabeads. High levels of Tax were retained on the HTLV-1 promoter and on the region of *RNASET2* gene encompassing +1069 to +1545 with respect to the predicted transcription start site. In contrast, substantially less Tax was retained on the *DKK1* promoter, which was negative for Tax in ChIP assays (Figure 1d). The nuclear extract used was prepared from SLB-1 cells, which showed specific binding of Tax to the HTLV-1 promoter and the *RNASET2* gene (Figure 1e).

**Figure 2.**
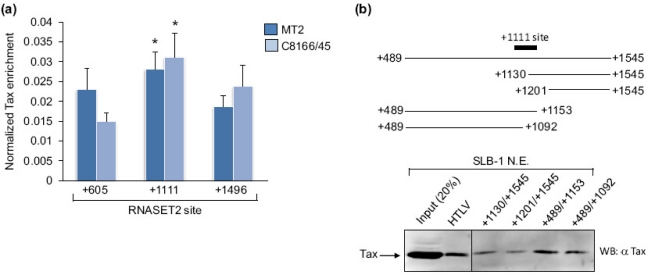
Levels of Tax enrichment at various regions of the *RNASET2* gene. (**a**) Levels of Tax enrichment at *RNASET2* gene sites centered at +605, +1111 and +1496 were determined by qPCR analysis of ChIP samples from MT-2 and C8166/45 cells. For each ChIP assay, the 2^CT(Input)−CT(IP)^ values for the three sites were added. The sums were then normalized to 1, and the 2^CT(Input)−CT(IP)^ values from each amplicon of a ChIP were multiplied by the normalization factor for that ChIP. The graph shows data from six and four ChIP assays for MT-2 and C8166/45 cells, respectively. Error bars show standard deviations. The asterisk denotes significantly higher enrichment of Tax at the +1111 site according to a Scheffé *post hoc* comparison preceded by ANOVA: *F*(2,15) = 9.69, *p* < 0.05 for MT-2; *F*(2,9) = 11.33, *p* < 0.05 for C8166/45. (**b**) Depicted are the *RNASET2* gene from +489 to +1545, the promoter deletion templates that were tested for Tax-binding, and the relative position of the +1111 amplicon that exhibited peak Tax-enrichment in ChIP assays. The Western blot shows Tax from an SLB-1 cell nuclear extract retained on immobilized DNA templates encompassing the different regions of the *RNASET2* gene.

The region of the *RNASET2* gene where Tax binds was further defined in MT-2 and C8166/45 cells using ChIP assays followed by qPCR amplification of three separate portions of the gene. The regions tested were centered at +605, +1111 and +1496 with respect to the RNA start site. The highest Tax-enrichment was observed at the +1111 site (Figure 2a). Because this approach provided a low resolution picture of where Tax is recruited on the promoter, we also used the immobilized template-binding assay to compare levels of Tax retained by various fragments of the *RNASET2* gene. Stronger Tax signals were detected using fragments encompassing +489 to +1153 and +489 to +1092 of the gene than those spanning +1130 to +1545 and +1201 to +1545 (Figure 2b). In Figure 1B, Tax was retained on a *RNASET2* promoter fragment encompassing +1069 to +1545. Therefore, the overall results from these assays suggest that Tax is recruited to the region from +1069 to +1092. This site is within the translated portion of the gene (the translational start site is at +408). An analysis of this DNA sequence using MatInspector [29] (Genomatix.de) revealed *cis*-elements for PRDM1/Blimp-1, NK6 and NKX homeodomain factors, and MYT1, but not for any known Tax-binding partners.

### 2.2. Tax Represses Expression of RNase T2

**Figure 3.**
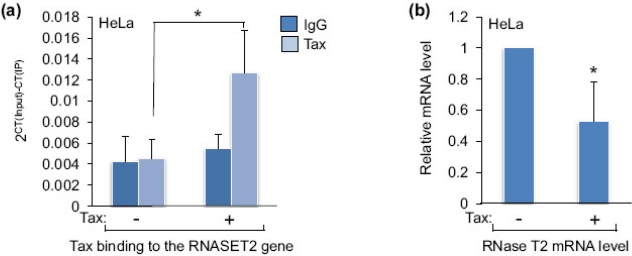

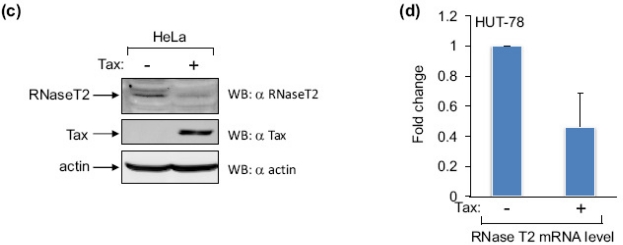
The effect of Tax on RNase T2 expression. (**a**) Recruitment of Tax to the *RNASET2* gene was assessed by comparing levels of Tax enrichment at the promoter in HeLa cells transfected with the pSG-5 empty vector (−) or the pSG-Tax expression vector (+). The graph shows the average 2^CT(Input)−CT(IP)^ values obtained for rabbit preimmune serum (IgG; used as a negative control) and for the Tax ChIP from five independent transfection/ChIP assays. Error bars show standard deviations. The asterisk denotes a *p*-value of 0.04 for a two-tailed Student *t*-test. (**b**) Relative levels of RNase T2 mRNA from HeLa cells transfected with pSG-5 (−) and pSG-Tax (+) were assessed by qRT-PCR. The graph shows the average 2^−ΔΔCT^ values from six independent transfection assays. The error bar shows the standard deviation. No standard deviation is shown for pSG-5, as it was used as the control treatment and set to 1 to show a relative difference in the mRNA level of the experimental treatment (pSG-Tax). The asterisk denotes a *p*-value of 0.009 for a two-tailed Student *t*-test. (**c**) The protein levels of RNase T2, Tax and actin from HeLa cells transfected with pSG-5 (−) or pSG-Tax (+) were assessed by Western blot. (**d**) Relative levels of RNase T2 mRNA from Hut-78 cells transfected with pSG-5 (−) or pSG-Tax (+) were assessed by qRT-PCR. The graph shows the average 2^−ΔΔCT^ values from two independent transfection assays.

To determine whether Tax alters the level of RNase T2 expression, we transfected HeLa cells with an expression vector for Tax or with the empty vector. In ChIP assays we found that Tax expression leads to enrichment of the viral protein over background detection at the *RNASET2* gene (Figure 3a), confirming that Tax is recruited to this site in these cells. This effect coincided with a decrease in the level of RNase T2 mRNA and protein (Figures 3b and 3c, respectively), indicating that Tax represses expression of this gene. Similar results were obtained using Hut-78 cells (Figure 3d), suggesting a similar effect in T-cells.

**Figure 4.**
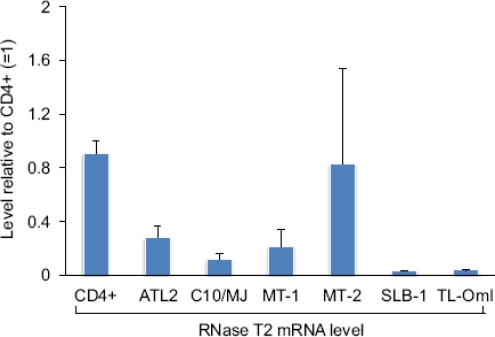
Levels of RNase T2 mRNA among HTLV-1-infected T-cell lines. Relative levels of RNase T2 mRNA from the cell lines and normal CD4+ T-cells were assessed by qRT-PCR. The graph shows the average 2^−ΔΔCT^ values from two independent RNA extractions. Levels were normalized to that from one of the CD4+ T-cell specimens (set to 1). Error bars show standard deviations.

We also compared relative levels of RNase T2 mRNA among a panel of HTLV-1-infected T-cell lines with that of CD4+ T-cells. We found that RNase T2 expression was reduced in five of the six cell lines tested (Figure 4). Surprisingly, MT-2 cells did not show a decrease in the transcript level compared to CD4+ T-cells. It is possible that Tax is ineffective in repressing RNase T2 expression when it is weakly associated with the gene, as suggested by ChIP data from MT-2 cells shown in Figure 1a. Interestingly, TL-OmI cells exhibited a low level of RNase T2 mRNA even though they do not express Tax. This observation may coincide with findings that the NF-κB pathway remains constitutively active in ATL cells lacking Tax [30,31], suggesting that some effects of Tax are retained in the transformed cells after expression of the viral protein is lost. Importantly, TL-OmI cells (and ATL-2 cells) are derived directly from an ATL patient.

### 2.3. Brm1, HDAC1 and Tax Exhibit Overlapping Patterns of Enrichment on the RNASET2 Gene

**Figure 5.**
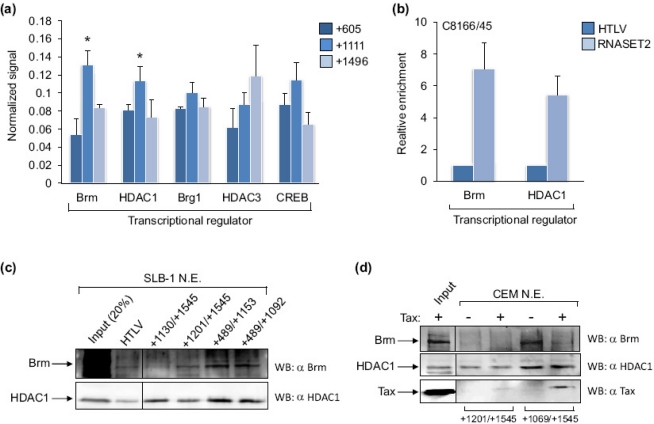
The association of Brm and HDAC1 with the *RNASET2* gene and the HTLV-1 promoter. (**a**) Levels of Brm, HDAC1, Brg-1, HDAC3 and CREB enrichment at sites centered at +605, +1111 and +1496 were determined by qPCR analysis of ChIP samples from MT-2, C8166/45 and SLB-1 cells. For each ChIP assay, the 2^CT(Input)−CT(IP)^ values for the three amplicons were added. The sums were then normalized to 1, and the 2^CT(Input)−CT(IP)^ values from each amplicon of a ChIP were multiplied by the normalization factor for that ChIP. Error bars show standard deviations. Asterisks denote significantly higher enrichment of Tax at the +1111 site according to a Scheffé *post hoc* comparison preceded by ANOVA: *F*(2,6) = 26.38, *p* < 0.01 for Brm (tested in C8166/45 cells); *F*(2,9) = 11.87, *p* < 0.05 for HDAC1 (tested in C8166/45 and SLB-1 cells). Data for CREB represent the average normalized enrichment from three independent ChIP assays using C8166/45 and MT-2 cells. Data for Brg1 and HDAC3 represent the average normalized enrichment from two ChIP assays using C8166/45 and MT-2 cells. (**b**) Levels of Brm and HDAC1 associated with the HTLV-1 and *RNASET2* genes in C8166/45 cells were determined by qPCR analysis of ChIP samples. The 2^CT(Input)−CT(IP)^ value obtained with primer sets for the *RNASET2* +1111 site was normalized to the value obtained with the HTLV-1 primer set, which was set to 1. The graph shows data averaged from two independent ChIP assays for each protein. Error bars show standard deviations. (**c**) The Western blots show Brm and HDAC1 from an SLB-1 cell nuclear extract retained on immobilized DNA templates encompassing the different regions of the *RNASET2* gene. (**d**) The Western blots show Brm and HDAC1 from a CEM nuclear extract retained on the indicated *RNASET2* gene templates in the absence and presence of recombinant, purified Tax.

To gain insight into how Tax associates with, and regulates transcription from the *RNASET2* gene, we used ChIP assays to identify proteins that colocalize to the same region of the gene as Tax. Because the DNA region targeted by Tax did not contain *cis*-elements for transcription factors known to associate with Tax, we focused mainly on interacting partners that repress transcription and do not bind directly to DNA. Protein-binding patterns at the *RNASET2* gene +605, +1111 and +1496 sites are shown in Figure 5a. In order to compare these patterns, we equalized the signals for the proteins represented in the graph (see legend), as the signal varied among the different antibodies used for the ChIP assays. A pattern matching that for Tax was observed with Brm, which is one of two interchangeable catalytic subunits of the SWI/SNF chromatin remodeling complex [32]. Interestingly, the homologous subunit, Brg-1, did not exhibit the same pattern of binding although it was this subunit that was isolated in a protein complex with Tax [33]. The histone deacetylase, HDAC1, also showed binding similar to Tax, while HDAC3 did not. Although these proteins have overlapping functions and interact with Tax [34,35], they are frequently found in separate repressor complexes [36]. CREB did not exhibit significant enrichment at the +1111 site compared to the flanking sites. This result was expected, as the +1111 site does not carry a consensus CRE, and CREB plays a positive role with Tax in activation of HTLV-1 transcription [5]. Signals for Brm and HDAC1 were significantly greater at the *RNASET2* +1111 site than at the HTLV-1 promoter (Figure 5b). In an immobilized template-binding assay, Brm and HDAC1 bound more efficiently to the +489/+1153 and +489/+1092 fragments of the *RNASET2* gene than the +1130/+1545 and +1201/+1540 templates, which parallels results obtained for Tax (Figure 5c). To determine whether Tax mediated the recruitment of these proteins to the *RNASET2* gene, we performed immobilized template-binding assays using nuclear extract derived from uninfected T-cells (CEM cells) and supplemented some reactions with recombinant, purified Tax (Figure 5d). We found that Brm and HDAC1 were efficiently retained on the template containing the target site for Tax in the absence of the viral protein. Addition of Tax did not augment binding of these proteins to the template, suggesting that Tax does not play a role in the recruitment of Brm and HDAC1 to *RNASET2* gene.

## 3. Experimental Section

### 3.1. Cell Culture, Transfection and Western Blot Analysis

T-cell lines were cultured in Iscove’s modified Dulbecco medium (IMDM) supplemented with 10% fetal bovine serum, 2 mM L-glutamine, 100 U/mL penicillin, and 50 μg/mL streptomycin. HeLa cells were cultured in Dulbecco’s modified Eagle’s medium (DMEM) supplemented as above. HeLa cells were transfected by electroporation as described [37], using cotransfection of pMACS 4.1 and the MACSelect system (Miltenyi Biotec) to purify transfected cells [38]. The expression vectors used in this study were pSG-5 and pSG-Tax [39]. HUT-78 cells were transfected by electroporation using a Gene Pulser Xcell (Bio-Rad) with 1 × 10^7^ cells in 675 uL RPMI/10 mM dextrose/0.1 mM dithiothreitol and 20 μg plasmid DNA (3:1 stiochiometric ratio of the expression vector of interest to pMACS K^k^.II) per 0.4 cm cuvette. Each cuvette was subjected to a single exponential decay pulse of 300 V/250 μF. Four cuvettes were pulsed per vector. Electroporated cells were cultured 48 h. Live cells were collected by centrifugation on Ficoll-Paque PLUS (GE Healthcare) as described by the manufacturer, and transfected cells were purified using the MACSelect system. Western blots were performed as described [40].

### 3.2. Chromatin Immunoprecipitation (ChIP) Assays and Microarray Analysis

ChIP assays for real-time PCR analysis were performed as described [41]. For some assays, cell lysates were sonicated using a Misonix Sonicator 4000 by pulsing samples in cold water in a 5.5 inch cup horn 15 times at an amplitude of 60 for 20 s with 30 s rests between pulses. For the microarray analysis, the ChIP assay was altered by replacing salmon sperm DNA with 0.5 mg/mL tRNA (Invitrogen) for the immunoprecipitation step. The total DNA from a single immunoprecipitation (IP) and 10 ng of input DNA were processed and amplified by ligation-mediated PCR as described [42]. Aminoallyl-dUTP was randomly incorporated into the DNA and coupled with the Cy3 (input) or Cy5 (IP) fluorescent dye as described [42]. Samples were combined to probe 12.2 K CpG island microarray (University Health Network Microarray Center, Princess Margaret Hospital, Ontario Cancer Center, Canada) [43], using hybridization conditions described [44]. Data was analyzed as described [42]. Antibodies against Brg-1 (sc-10768), Brm (sc-6450), HDAC1 (sc-7872) and HDAC3 (sc-11417) were from Santa Cruz Biotechnology. The antibody against CREB was from Millipore (06-863). The antibody against Tax was from the NIH AIDS Research and Reference Reagent Program (hybridoma 168B17–46-92).

### 3.3. Real-Time PCR

Real-time PCR and subsequent analyses were performed as described [28]. The following primers were used for ChIP samples:

vCRE-F, 5′-ATCATAAGCTCAGACCTCCGGGAA; vCRE-R,
5′-CCTGAGGACGGCTTGACAAACAT; Gag-F, 5-CGCTCATCACTGGCTTAACTTCCT; Gag-R,
5′-TGGTGGAAATCGTAACTGGAGGGA; RNaseT2+1496F,
5′-CGGTGCTGCAAAGAACTCCAACTT; RNaseT2+1496R,
5′-TCCTATTAACCCGATCATTACCCA; RNaseT2+1111F,
5′-AGCCAAGGGAAGGGATTCAGAACA; RNaseT2+1111R,
5′-TAATTTCAGCTTTCACTGCGGCGG; RNaseT2+605F,
5′-CGCTGCTCAACTTCCCAACTTCTT; RNaseT2+605R,
5′-CCCTAGAGCTTTCCACTGGGTGAA; DKK1-331F and DKK1-331R [28].

RNA extraction, cDNA sythesis, qRT-PCR and data analysis were performed as described [28]. The following primers were used for cDNA samples: RT-RNaseT2F, 5′-ACTGGCCTGACGTAATTCACTCGT; RT-RNaseT2R, 5′-TGTAGAGTTCCAGGCTTCTGCCAA; UBE2D2-F and UBE2D2-R [28].

### 3.4. Immobilized Template-Binding Assays

Assays were performed essentially as described [45]. For each binding reaction, 2 pmol of a biotinylated DNA fragment was bound to 10 μL of M-280 streptavidin Dynabeads (Invitrogen) as described by the manufacturer and blocked in 500 μL ITB (0.1 M KCl, 20 mM HEPES [pH 7.9], 1 mM MgCl_2_, 20 μM ZnSO_4_, 10% glycerol, 0.01% Triton X100, 0.5 mM EDTA, 0.5 mM PMSF, 1 mM benzamidine, 5 mM DTT) with 5% BSA for 1 h at 4 °C. The blocked DNA-coupled beads were cleared, combined with 250–500 μg nuclear extract in a total volume of 500 μL of ITB/5% BSA, and mixed for 2 h at 4 °C. Nuclear extract was prepared from SLB-1 and CEM cells as described [46]. Tax was expressed and purified as described [47]. Tax (90 pmol) was preincubated with nuclear extract for 1 h at 4 °C prior to adjusting reactions to 500 μL of ITB/5% BSA. Bead complexes were washed extensively with ITB. Bound proteins were resolved by SDS-PAGE and detected by Western blot using the same antibodies as those used for ChIP assays.

## 4. Conclusions

The oncogenic effects of Tax originate, in part, from its ability to deregulate expression of numerous genes. In some cases, Tax mediates changes in transcription directly from the cellular promoter [6,48–50]. In this study, we have identified such a situation. A screen for Tax-binding sites through a microarray analysis of ChIP samples identified the *RNASET2* gene as a site targeted by the viral protein, and subsequent data supported this finding. Although Tax is often classified as a transcriptional activator, we found that expression of RNase T2 was repressed by the viral protein. This effect is not unique, as Tax has been reported to downregulate transcription of other cellular genes, including those encoding c-Myb, cyclin A, cyclin D3, α-polymerase, β-polymerase, Lck, p53, p18ink4C, SHP-1 and Znf268 [6,7,11,49,51–54].

Mechanisms of Tax-mediated repression that have emerged involve Tax interactions with CREB, p300/CBP, TAX1BP1, and HDAC1 [6–8,34,55]. Only repression through CREB and HDAC1 has been shown to occur through association of Tax with the target promoter *in vivo* [6,49]. On the *RNASET2* gene, it is unlikely that CREB plays a role in repression, as CRE-like elements are absent from the Tax-binding region of the promoter, and CREB did not exhibit peak enrichment at this site. In contrast, HDAC1 and Brm did show a similar pattern of enrichment on the gene as Tax. However, their association with the gene does not appear to require Tax. Because Tax interacts with HDAC1 and multiple Brg-1 associated factors that form a complex with Brm [33,34], it is possible that these proteins assist in the recruitment of Tax to the *RNASET2* gene. Although the mechanism of repression remains unclear, it is interesting that repression of Znf268 expression also involves recruitment of Tax within the transcribed portion of the gene [6]. It is therefore possible that, when bound to these downstream sites, Tax hinders transcriptional elongation. Further work is needed to test this hypothesis.

The inhibitory effect of RNase T2 on tumor growth is largely seen in the context of the tumor microenvironment using a mouse model [26]. Therefore, how this protein functions as a tumor suppressor remains to be determined. In HTLV-1-infected cells, it is possible that reduced RNase T2 expression by Tax helps prevent these cells from undergoing senescence or apoptosis due to other effects of the viral protein. Among its pleiotropic effects, Tax increases the prooxidant state within cells, which can lead to senescence or apoptosis [56,57]. Recently, the yeast paralogue of RNase T2 was shown to help mediate an apoptotic response to oxidative stress [58]. Therefore, in conjunction with higher levels of thioredoxin found in cells expressing Tax [59], concomitant lower levels of RNase T2 may be important for survival and proliferation.
